# Social Classification: The Need to Update in the Present Scenario

**DOI:** 10.4103/0970-0218.39245

**Published:** 2008-01

**Authors:** AK Agarwal

**Affiliations:** Department of Community Medicine, G. R. Medical College, Gwalior - 474 009, (MP), India

## Introduction

Socioeconomic Status (SES) is an important determinant of the standard of living and health status as it influences the incidence and prevalence of various health conditions. Socioeconomic status also influences social security in terms of the accessibility, affordability, acceptability and actual utilization of various health facilities.

The need for developing a uniform system of socioeconomic classification of the population universally based on the income with scientific basis and should applied with ease and simplicity in each sector or strata wise of population.

There have been several attempts to develop different scales to measure socioeconomic status but Prasad's classification (1961)(1) based on the per capita monthly income and later modified in 1968(2) and 1970,(3) has been extensively used in the Indian scenario and has been quite effective in performing the t task under discussion. But with the passage of time and due to the inflationary trends in the economy, the original income limits set in these classifications have become substantially low and impractical. Realizing this need, Kumar(4) has updated the classification linked with the All India Consumer Price Index (AICPI) which has also become impractical today and has lower validity due to great variations in the Consumer Price Index.

By 1993–1994 however, the inflation rate was governed by the All India Whole Price Index series,(5) creating an urgent need to link Prasad's classification (1961) with the All India Whole Price Index whereas Prasad's (1961) classification has taken for a basis of modification in terms of latest scenario of cost of living index (COLI). In order to solve this problem, a hypothetical value (0.53)(6) has been developed in relation to the base year of 1993-1994 when the new series of the All India Wholesale Price Index started.(5,7)

The hypothetical value was calculated based on the concept of the cost-of-living index (COLI), which pertains to the WPI in India. As the COLI is not directly observable, the WPI employs a number of formulae that offer approximations to the measurement objectives. WPI uses the Laspeyres formula to average the price changes due to inflation across different categories of items, because COLI for the each current month is based on the cost of this month's market prices for the items used by the community may be changed due to inflation in wholesale price, of achieving the standard of living actually attained in the base period.(8,9)

The ratio of this hypothetical cost to the actual cost of the base period is the lowest expenditure level necessary at this month's price to achieve the base period's living standard. This framework of WPI and the inflation rate from the base period has guided and will continue to guide operational decisions about the construction of the index.(8,9)

It is a simple method of multiplying the income limits of classification with a multiplication factor and rounding off the values to the nearest rupee. Multiplying the AIWPI at the time of study by the hypothetical value could help to derive the multiplication factor.

Therefore, the multiplication factor = Value of AIWPI(10) × 0.53

The next step is to multiply Prasad's income limits by the multiplication factor. There is one more class-‘Below Poverty Line’ (BPL) included by the author because this concept of Below Poverty Line was not started in 1961.

Income limits thus obtained, are far more practical and realistic. For example, to compute a social classification for December 2004, the multiplication factor will be = 189.2(10) × 0.53 = 100.27 or 100.

The proposed classification for this period is given in Table 1.

**Table 1:** Proposed social classification for the month of December 2004

Social class	Per capita monthly income limits (Rs.)	
	
	Prasad's classification	Modified proposed
	Classification for the month of December 2004	
I. Upper high	100 and above	10000 and above
II. High	50-99	5000-9999
III. Upper middle	30-49	3000-4999
IV. Lower middle	15-29	1500-2999
V. Poor	Below 15	500-1499
VI. Very poor or		
Below poverty		
line (BPL)	-	Below 500

As such the value of the AIWPI has not varied according to the geographical area or work forces and urban or rural conditions do not influence the classification. Hence, the value of the AIWPI can be used safely and are regularly published in various weekly fortnightly financial newspapers and magazines.

## References

[1] Prasad BG (1961). Social Classification of Indian families. J Indian Med Assoc.

[2] Prasad BG (1968). Social Classification of Indian families. J Indian Med Assoc.

[3] Prasad BG (1970). Changes proposed in Social classification of Indian families. J Indian Med Assoc.

[4] Kumar P (1993). Social classification-need for constant updating. Indian J Community Med.

[5] Hindu Business Line (2004).

[6] Economic Advisor (2001).

[7] Kumar R, Mehndiratta D (2004). Hindu Business Line.

[8] (1974). A conceptual framework for the revised price index. Proceedings of the business and economic statistics section.

[9] (1998). Measurement issues in the price index. Statistical Journal of the United Nations, ECE 15.

[10] Hindu Business Line (2005).

